# Real-world monitoring progress towards the elimination of hepatitis C virus in Australia using sentinel surveillance of primary care clinics; an ecological study of hepatitis C virus antibody tests from 2009 to 2019

**DOI:** 10.1017/S0950268821002624

**Published:** 2021-12-06

**Authors:** Anna Lee Wilkinson, Alisa Pedrana, Michael W. Traeger, Jason Asselin, Carol El-Hayek, Long Nguyen, Victoria Polkinghorne, Joseph S. Doyle, Alexander J. Thompson, Jessica Howell, Nick Scott, Wayne Dimech, Rebecca Guy, Margaret Hellard

**Affiliations:** 1 Disease Elimination Program, Burnet Institute, Melbourne, Australia; 2 School of Public Health and Preventive Medicine, Monash University, Melbourne, Australia; 3 Department of Infectious Diseases, The Alfred and Monash University, Melbourne, Australia; 4 Department of Gastroenterology, St Vincent's Hospital, Melbourne, Australia; 5 Department of Medicine, University of Melbourne, Melbourne, Australia; 6 National Serology Reference Laboratory, Melbourne, Australia; 7 Kirby Institute, UNSW, Sydney, Australia; 8 Doherty Institute and Melbourne School of Population and Global Health, University of Melbourne, Melbourne, Australia

**Keywords:** Hepatitis C, people who inject drugs, primary care, surveillance

## Abstract

To achieve the elimination of the hepatitis C virus (HCV), sustained and sufficient levels of HCV testing is critical. The purpose of this study was to assess trends in testing and evaluate the effectiveness of strategies to diagnose people living with HCV. Data were from 12 primary care clinics in Victoria, Australia, that provide targeted services to people who inject drugs (PWID), alongside general health care. This ecological study spanned 2009–2019 and included analyses of trends in annual numbers of HCV antibody tests among individuals with no previous positive HCV antibody test recorded and annual test yield (positive HCV antibody tests/all HCV antibody tests). Generalised linear models estimated the association between count outcomes (HCV antibody tests and positive HCV antibody tests) and time, and *χ*^2^ test assessed the trend in test yield. A total of 44 889 HCV antibody tests were conducted 2009–2019; test numbers increased 6% annually on average [95% confidence interval (CI) 4–9]. Test yield declined from 2009 (21%) to 2019 (9%) (*χ*^2^*P* = <0.01). In more recent years (2013–2019) annual test yield remained relatively stable. Modest increases in HCV antibody testing and stable but high test yield within clinics delivering services to PWID highlights testing strategies are resulting in people are being diagnosed however further increases in the testing of people at risk of HCV or living with HCV may be needed to reach Australia's HCV elimination goals.

## Introduction

The availability of direct-acting antivirals (DAAs) for the treatment of hepatitis C virus (HCV) shifted Australia's public health efforts to target the elimination of HCV by 2030. The cost of DAAs was subsidised through Australia's universal healthcare system (Pharmaceutical Benefits Scheme; PBS) from 1 March 2016 in Australia [1]. Critically, primary care practitioners can prescribe DAAs and treatment is available to anyone with chronic HCV, regardless of disease stage, including people who inject drugs (PWID). The broad availability of subsidised DAAs in Australia means the goal of eliminating HCV relies on the timely diagnosis of people with active HCV infection to improve health outcomes for individuals and prevent onward transmission. The PBS listing resulted in an immediate and significant uptake of treatment; overall an estimated 82 000 people were treated with DAAs between January 2016 and December 2019, representing ~43% of all people estimated to be living with HCV in 2015 [2]. However, much of the treatment uptake occurred in 2016 (32 877 treatment initiations) followed by a marked decline in annual numbers treated; [3] in 2019, approximately 11 580 DAA treatment initiations occurred [2]. Declines in DAA initiation by specialists occurred as expected, but an anticipated increase in treatment by general practitioners in the context of DAA prescribing guidelines has not eventuated [2].

Mathematical modelling suggests for Australia to reach its elimination targets, additional efforts are needed to ensure those undiagnosed are identified and engaged in care, and those previously diagnosed are re-engaged in care [4]. Australia's National Hepatitis C Strategy includes a specific target of increasing the proportion of people living with hepatitis C who are diagnosed to 90% [5]. Modelling by Scott *et al*. [4] suggests that elimination efforts in Australia now need to focus on increasing the uptake of HCV testing, particularly among PWID. Australia's National Hepatitis C Testing Policy outlines that hepatitis C diagnosis begins with an immunoassay for HCV antibody detection as a screening test. Priority populations for hepatitis C testing include people who have previously or are currently injecting drugs, people born in countries with intermediate to high HCV prevalence, and people who have been incarcerated [6]. In this context, testing strategies must ensure testing is efficient and targeted at those at risk of HCV infection whilst avoiding unnecessary testing in those with minimal infection risk. Testing strategies also need to facilitate an individual's timely link to treatment if positive, improving their health and stopping onward HCV transmission.

Population-level monitoring of HCV diagnostic testing and outcomes can be used to evaluate efforts to expand access to DAAs and prevent transmission, including treatment-as-prevention strategies. With ongoing testing and reduced transmission through treatment-as-prevention, the number of undiagnosed people would be expected to decrease over time, and hence the HCV antibody test yield (the proportion of tests returning a positive result among individuals without a previous positive test) would also be expected to decrease. Observing no change to test yield over time would indicate either an increase in testing efficiency (i.e., improved or adaptive targeting of testing), or that the undiagnosed target population is approximately stable. Current Australian evidence of trends in HCV antibody positivity is confined largely to serial cross-sectional sero-surveys of attendees at needle and syringe programmes [7] and notifications to jurisdictional health departments of positive HCV antibody tests [8]. The Australian Needle Syringe Program Survey reported recent declines in HCV antibody positivity from 57% in 2015 to 45% in 2019 [7]. Also notifications of hepatitis C (2009–2018) peaked in 2016 (12 739 notifications) followed by a decline to 9493 in 2018 [8].

Whilst valuable data, neither notifications nor cross-sectional surveys show how testing practices may be influencing case-finding success. Trends in testing and case-finding are important indicators for evaluating efforts to eliminate HCV, particularly in the context of expanded access to treatments in primary care, where most HCV diagnostic testing has historically occurred. This study used data from a sentinel surveillance system of primary care clinics in Victoria, Australia, that provide targeted services for PWID. The study aimed to (1) describe the trends over time in HCV antibody tests conducted and (2) examine the efficiency of HCV testing by reporting HCV antibody test yield.

## Methods

### Setting

ACCESS is a sentinel surveillance system for blood-borne viruses (BBV; HIV, hepatitis B virus, HCV) and sexually transmissible infections (STIs; chlamydia, gonorrhoea, syphilis). The aim of ACCESS is to monitor the testing, diagnosis and management of these infections and evaluate the impact of relevant health interventions, thus informing Australia's strategic response to BBVs and STIs [9]. ACCESS has targeted recruitment of health services with higher caseloads of patients living with or at greater risk of acquisition of BBVs and STIs, with consideration given to the system's coverage nationally and within each state and territory. General practice and community health clinics that specialise in the health of people who currently inject or previously injected drugs were included in this analysis. These clinics provide a range of specialised services such as onsite needle and syringe programmes, have one or more prescribers of opiate agonist therapy (OAT), and promote and provide viral hepatitis testing, linkage to care and/or treatment. These clinics also provide general health services to PWID and the broader community.

### Data collection

Data are extracted from databases at participating sites using specialised health data extraction software known as GRHANITE^TM^ [10]. GRHANITE was developed by the Health and Biomedical Informatics Centre's Unit at the University of Melbourne (www.grhanite.com) and is used to routinely extract retrospective, line-listed data from patient management systems at participating ACCESS sites [9, 11] Prior to their extraction, each patient record is allocated a unique hashed record identifier and linkage keys generated from encrypting patient identifying information. The following patient identifiers are used to create the linkage key but are not extracted from the patient management system by ACCESS: five digits of the Medicare number, date of birth, sex, first name, last name, and residential code. Once extracted, linkage keys are used to probabilistically link de-identified patient records both between and within ACCESS sites.

### Analysis

Data from 12 clinics located in Victoria were included in this ecological study. To describe trends in testing practices that enhance detection of new diagnoses, test records from 1 January 2009 to 31 December 2019 among individuals with no record of a previous positive HCV antibody test were included; all 12 primary care clinics consistently provided data for the study period. All negative HCV antibody tests and an individual's first positive HCV antibody test on or before 31 December 2019 were included. We report an annual number of HCV antibody tests (overall and by negative and first positive result). To examine the efficiency of HCV antibody testing practices, we report annual HCV antibody test yield (positive HCV antibody tests divided by the total number of HCV antibody tests (positive and negative) in a calendar year. Overall trends in annual numbers of HCV antibody tests conducted and the number of HCV antibody positive tests were estimated using a generalised linear model. The trend in test yield over time was estimated with a *χ*^2^ test for trend.

Detailed information on individuals' HCV risk and/or clinical indication for an HCV antibody test was not available, however variables of age (calculated from the year of birth), sex (male and female), and history of an electronic prescription for OAT of individuals were available. To estimate HCV antibody testing and test yield among sub populations that differ in HCV risk, analyses were stratified by age group and sex, and a sub-analysis of individuals that were prescribed OAT. Individuals with at least one prescription for OAT in a calendar year were assigned as having received OAT in that year.

### Ethics

Ethics approval for the ACCESS was provided by the Alfred Hospital Human Research Ethics Committee (Project 248/17), as well as several specialised committees for key populations, including ACON, Thorne Harbour Health, and the Aboriginal Health and Medical Research Council. As our study analyses de-identified data collected under the auspices of public health surveillance, individual patient consent was not required. Individuals can opt-out of the ACCESS surveillance system.

Analysis was conducted in Stata (version 17, StataCorp, College Station, Texas) and R (version 4.0.2).

## Results

**Fig. 1.**
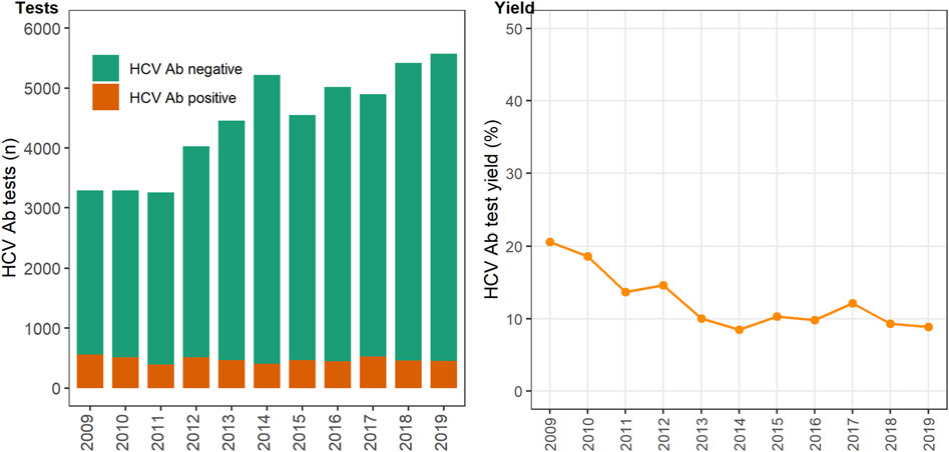
Number of HCV antibody negative and positive tests and test yield by year, Victoria, Australia, 2009* to 2019, *N* = 44 889 HCV antibody tests. Figure 1. Footnote: *2009 and 2010 have inflated test yield; because ACCESS commenced data collation in 2009, these years are less likely to have negative HCV antibody tests contributing to the denominator. Ab: antibody.

In 12 primary care clinics that specialise in the health of PWID a total of 44 889 HCV antibody tests were undertaken between 2009 and 2019 (Table 1), with the total number of tests (negative and positive) testing increasing on average each year by 6% (test for overdispersion: *P* = 0.005, *α* = 51.2; IRR 1.06; 95% CI 1.04–1.09). The number of positive HCV antibody tests remained stable ~450–500 per annum (test for overdispersion *P* = 0.02, *α* = 3.6; IRR 0.99; 95% CI 0.97–1.01; Fig. 1). Overall, test yield declined between 2009 and 2019 (*χ*^2^ *P* = <0.01). Test yield was highest in 2009 and 2010, although this is expected because individuals have less opportunity to contribute repeat negative HCV antibody tests compared to later years, when repeat negative tests accumulate and repeat positives are excluded. Test yield then declined between 2011 and 2013 and remained relatively stable between 2013 and 2019 (Fig. 1).

**Table 1.** Number of HCV tests conducted, the number positive, the number negative and the HCV antibody test yield^a^, by year, Victoria, Australia, 2009 to 2019, *N* = 44 889 HCV antibody tests

Year	HCV antibody tests (*n*)	HCV antibody negative tests (*n*)	HCV antibody positive tests (*n*)	HCV antibody test yield (%)
2009	2728	2166	562	20.6^b^
2010	2776	2260	516	18.6^b^
2011	2863	2471	392	13.7
2012	3513	3000	513	14.6
2013	4639	4173	466	10.0
2014	4810	4403	407	8.5
2015	4552	4083	469	10.3
2016	4570	4122	448	9.8
2017	4365	3836	529	12.1
2018	4957	4496	461	9.3
2019	5116	4662	454	8.9

a 100 × (HCV antibody positive tests/ HCV antibody tests).

b Note that 2009 and 2010 have inflated test yield as ACCESS commenced data collation in 2009, therefore these years are less likely to have negative HCV antibody tests contributed to the denominator.

**Fig. 2.**
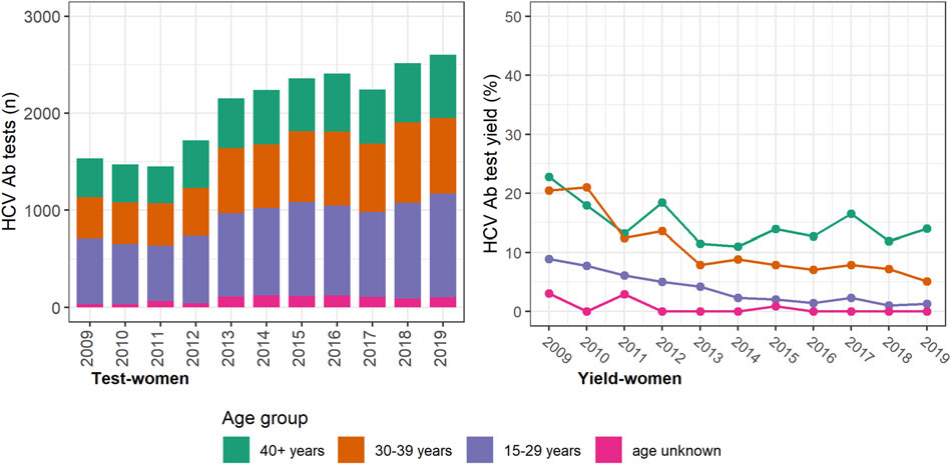
Number of HCV antibody tests and test yield among women by year and age groups, Victoria, Australia, 2009* to 2019, *n* = 22 639 HCV antibody tests among women. Figure 2. Footnote: *2009 and 2010 have inflated test yield; because ACCESS commenced data collation in 2009, these years are less likely to have negative HCV antibody tests contributing to the denominator. Ab: antibody.

**Fig. 3.**
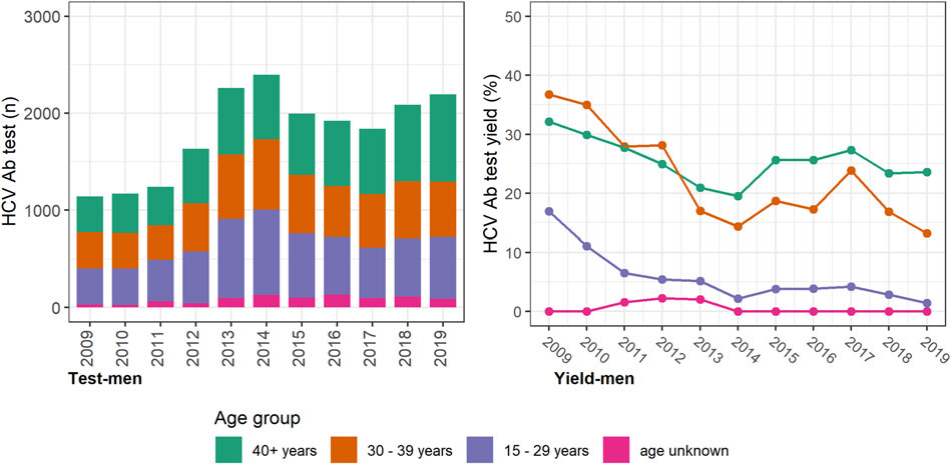
HCV antibody tests and test yield among men by year and age groups, Victoria, Australia, 2009* to 2019, *n* = 19 869 HCV antibody tests among men. Figure 3. Footnote: *2009 and 2010 have inflated test yield; because ACCESS commenced data collation in 2009, these years are less likely to have negative HCV antibody tests contributing to the denominator. Ab: antibody.

**Fig. 4.**
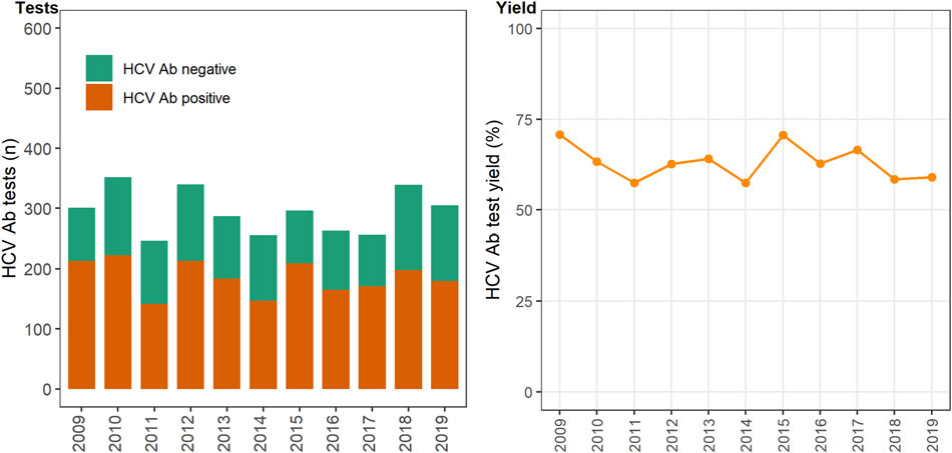
Number of HCV antibody negative and positive tests and test yield among individuals with at least one electronic prescription for OAT, by year, Victoria, Australia, 2009–2019, *N* = 3243 HCV antibody tests. Figure 4. Footnote: *2009 and 2010 have inflated test yield; because ACCESS commenced data collation in 2009, these years are less likely to have negative HCV antibody tests contributing to the denominator. Ab: antibody.

The annual number of HCV antibody tests conducted was consistently higher among women, with most occurring among women <40 years and a prominent increase seen between 2012 and 2013 (Fig. 2). However, the test yield was consistently higher among men and consistently higher among older men and women (⩾30 years; Figure 3 (men) and Figure 2 (women)). Among individuals with a history of an electronic prescription for OAT, the number of HCV antibody tests conducted annually remained largely unchanged between 2009 and 2019. Test yield remained >50% in this group, with a peak occurring in 2015 (Fig. 4).

## Discussion

Data from 12 primary care clinics participating in a sentinel surveillance system showed an overall increase in the number of HCV antibody tests conducted (2009–2019). Test yield declined over the observation period but from 2016 when DAAs were universally available the number of individuals who tested HCV antibody positive and the test yield remained stable. However, the overall test yield was high compared to a general population prevalence of ~1% [12] and was ~9% in 2019. Compared to men, more HCV antibody tests and lower test yield was observed among women <40 years; an unknown proportion of these tests are likely to be routine antenatal screening as Australia's Pregnancy Care Guidelines have recommended universal hepatitis C testing since 2016 [13]. Test yield was >20% among older men, consistent with data from HCV notifications [8] and surveys among attendees at needle and syringe programmes [7] Test yield was >50% among individuals with a history of a prescription for OAT. High test yields in primary care clinics highlight that activity within primary care clinics has been successful in identifying people exposed to hepatitis C. It is crucial for Australia's HCV elimination efforts to continue to promote and support hepatitis C testing in these specialist primary care clinics that provide services to PWID, especially to patients accessing OAT.

People currently injecting or who previously injected drugs are most at-risk of HCV in Australia, and are therefore prioritised in the public health response to HCV [5] and Australia's HCV testing policy [6]. Ideally, an increase in HCV antibody testing would have been observed among those with a history of OAT in our dataset, at least from 2016 when DAA treatments were able to be prescribed in primary care settings. The National Testing Policy further encourages reflex HCV RNA testing, therefore increases in HCV antibody testing are likely to result in increased diagnosis where reflex testing is undertaken [6]. Despite the benefits of people accessing HCV care in Australian primary settings, [14, 15] HCV treatment by primary care providers has not increased, [16] and significant gaps remain in linking those diagnosed in primary care to timely treatment initiation [17]. While we did not monitor trends in HCV treatment in these clinics, our analysis suggests there are ongoing and significant opportunities for HCV diagnoses, and therefore treatment in primary care clinics, especially to individuals accessing OAT. However, some studies suggest general practitioners may lack confidence in assessing HCV risk, completing diagnoses and initiating treatment, [18] alongside meeting the other primary, preventive, and complex care needs of patients. Primary care clinics may also require additional resources to increase HCV diagnosis and treatment given the competing priorities within primary settings, or, in the context of high service demand, may not be well-structured to pivot to HCV diagnoses and care as a priority. Health promotion efforts to ensure clinicians and the community are aware and continually reminded that HCV treatment is available, can be prescribed in primary care settings and can be prescribed to patients regardless of injecting drug history also remains a priority.

Trends in population-level testing and test yield in this study need to be interpreted cautiously. First, clinical and risk indications for testing or not testing were not available. Whilst sites were included in ACCESS because they provided services specific to PWID, clinics also provided general health services, including to people with no history of injecting drug use. Also, individuals' specific HCV risk, including a person's history of currently or previously injecting drugs. It is important to note that the population in this study represents people engaged with primary care services and individuals not engaged with primary care services remain an important target population for hepatitis C elimination. However, data from these sites remain important for assessing Australia's HCV response given HCV antibody test yield was substantially higher (>10% across the observation period) than the estimated population prevalence of HCV and very high among individuals with a history of OAT. Second, although individuals can be linked across ACCESS clinics with a unique identifier, episodes of care or HCV testing at clinics not in ACCESS would be missed. This study however included 11 years of prospective data from 12 primary care sites that are indicative of services that, in the context of simplified and highly tolerable DAA treatment and Australia prescribing guidelines, should be scaling up testing, case detection and treatment for HCV to help meet Australia's elimination targets. The study used absolute numbers of HCV tests pooled across 12 sites; specific changes within a practice or external factors such as changes in a community the clinics serves that may influence HCV antibody tests conducted are not captured in the data. However, the rich data set of HCV antibody testing over time provided by ACCESS also allowed for an analysis of trends in HCV antibody testing and positivity which has not previously been reported in Australia.

To achieve HCV elimination 2030 targets in Australia it is vital that testing levels increase and are maintained so there are enough people diagnosed and linked to HCV care and treatment to decrease the prevalence and incidence of HCV. This study suggests that there remain opportunities to increase HCV testing and diagnosis in primary care settings attended by high numbers of people who currently inject of who have a history of injecting drug use. The yield was high at these clinics, particularly in patients with a history of OAT. If Australia is to achieve HCV elimination a comprehensive approach is required that addresses barriers to presenting to primary care and to HCV testing for populations at-risk of HCV acquisition and transmission. Increases in the capacity of the primary care workforce will be needed, alongside tailored health promotion, to improve testing and treatment uptake in Australia, and in other settings looking to eliminate HCV.

## Data Availability

Deidentified patient data is available on request from ACCESS (https://accessproject.org.au/contact)
