# Real-world monitoring progress towards the elimination of hepatitis C virus in Australia using sentinel surveillance of primary care clinics; an ecological study of hepatitis C virus antibody tests from 2009 to 2019 – CORRIGENDUM

**DOI:** 10.1017/S0950268822000310

**Published:** 2022-03-04

**Authors:** Anna Lee Wilkinson, Alisa Pedrana, Michael W Traeger, Jason Asselin, Carol El-Hayek, Long Nguyen, Victoria Polkinghorne, Joseph S Doyle, Alexander J Thompson, Jessica Howell, Nick Scott, Wayne Dimech, Rebecca Guy, Margaret Hellard, Mark Stoové

**Affiliations:** 1 Disease Elimination Program, Burnet Institute, Melbourne, Australia; 2 School of Public Health and Preventive Medicine, Monash University, Melbourne, Australia; 3 Department of Infectious Diseases, The Alfred and Monash University, Melbourne, Australia; 4 Department of Gastroenterology, St Vincent's Hospital, Melbourne, Australia; 5 Department of Medicine, University of Melbourne, Melbourne, Australia; 6 National Serology Reference Laboratory, Melbourne, Australia; 7 Kirby Institute, UNSW, Sydney, Australia; 8 Doherty Institute and Melbourne School of Population and Global Health, University of Melbourne, Melbourne, Australia

The original version of this article was published with an author missing from the author list., Mark Stoové should have been included. This has now been corrected in the original version.
